# Safety, Sensory Quality and Nutritional Value of Hybrid Meat Products Made from Turkey Meat and Red Beans Preserved with a Bioprotective Culture

**DOI:** 10.3390/molecules30030691

**Published:** 2025-02-04

**Authors:** Małgorzata Karwowska, Patrycja Skwarek, Elżbieta Solska, Agata Nowaczyk, Andrzej Goławski, Przemysław Wojtaś, Dariusz M. Stasiak

**Affiliations:** 1 Sub-Department of Meat Technology and Food Quality, Department of Animal Food Technology, University of Life Sciences in Lublin, Skromna 8, 20-704 Lublin, Poland; patrycja.skwarek@up.lublin.pl (P.S.); elzbieta.solska@up.lublin.pl (E.S.); agata.nowaczyk@up.lublin.pl (A.N.); dariusz.stasiak@up.lublin.pl (D.M.S.); 2 Meat Plant Mościbrody sp. z o.o., Mościbrody 53, 08-112 Wiśniew, Poland; andrzej.golawski@moscibrody.pl (A.G.); p.wojtas@moscibrody.pl (P.W.)

**Keywords:** hybrid meat product, turkey meat, red bean, bioprotection

## Abstract

The current study assessed the quality and safety of hybrid meat products made from turkey meat and red beans with the addition of SAFEPRO^®^ B-LC-20 protective cultures. The tested materials were hybrid products produced with turkey thigh muscles and red beans in 100:0, 60:40, 50:50 and 40:60 ratios. During a 15-day storage period, research was carried out on the physicochemical and microbiological properties, antioxidant capacity, fatty acid profile and sensory characteristics. The results showed that the count of *Enterobacteriaceae* in hybrid meat products did not differ significantly depending on the formulation. The addition of red beans in a hybrid meat product formulation significantly increased the antioxidant activity of the products compared to a sample made of 100% meat. The samples with red beans were characterized by significantly lower values of n-6/n-3, UFA/SFA and PUFA/SFA compared to samples produced with turkey thigh muscles and red beans in a ratio of 100:0. In summary, the formulation combining turkey meat and beans in a ratio of 60:40 is recommended as optimal, enabling the creation of a safe hybrid meat product with properties similar to those of a full-meat product.

## 1. Introduction

In recent years, due to concerns surrounding the environmental and health impact of meat production and consumption, there has been increased interest in a flexitarian diet that involves limiting the consumption of meat and animal-based products without complete abstinence [1,2]. Flexitarians are actively trying to reduce their intake of animal products and are likely to consume alternative protein sources [3]. As related by Dagevos [3], there are multiple recent studies from various affluent countries revealing that flexitarianism is not a fringe behavior. As recent consumer research shows, in various high-income countries, segments of contemporary food consumers are changing their meat-eating behavior and restricting their intake. Consumers called flexitarians practice strategies that include both spreading meat consumption throughout the week and eating plant-based meals, as well as reducing meat portion sizes while increasing their consumption of plant-based proteins and vegetables [4]. In this context, hybrid meat products are emerging as an alternative to traditional meat products as they combine meat with plant-based ingredients (such as pulses, grains, fruits and vegetables in different ratios, from about 25% to about 50%). According to the unofficial definition, hybrid meat products contain plant-based ingredients that are not added as fillers but for their positive technological, nutritional and sensory properties [5,6]. Such products provide a meaty flavor and texture while also containing a range of plant-based ingredients, often with health-promoting properties (such as fiber, vitamins, and minerals), which can result in the products having functional properties. Hybrid meat products may, therefore, offer an interesting alternative for consumers who want to consume more plant-based ingredients, as well as those looking for new flavors and diverse nutritional profiles [7].

Red beans as raw materials can play a significant role in the development of hybrid meat products due to their nutritional and physicochemical properties. Kidney beans, as a legume, have the potential to provide health-promoting properties to meat products due to their high protein content, fiber, other bioactive compounds, and favorable amino acid composition. Legume proteins are characterized by a low content of sulfur amino acids but contain a large amount of the essential amino acid lysine [8]. In addition, soluble dietary fibers are one of the main bioactive components in legumes exhibiting various biological functions (e.g., antioxidant, anti-diabetic, prebiotic, and immunomodulatory activities) [9,10,11]. As shown by Wu et al. [9], soluble dietary fibers of legumes are primarily composed of complex polysaccharides, which are rich in pectic polysaccharides. Moreover, they display potential antioxidant, antiglycation, immunostimulatory and prebiotic activities. Jayamanohar et al. [12] have shown that dietary polysaccharides extracted from red kidney beans can stimulate the growth of probiotic strains, e.g., *Lactobacillus plantarum* and *L. fermentum*, which can also stimulate the growth of beneficial gut microbiota (e.g., *Bifidobacterium* spp. and *Lactobacillus* spp.) during in vitro batch fecal fermentation.

An important issue in the production of hybrid meat products is to ensure high quality and safety [13]. An innovative solution that contributes to enhancing the safety of hybrid meat products is the use of microorganism cultures in the field of bioprotection. Bioprotection, therefore, consists of introducing a culture of microorganisms and their metabolites into a food product in order to improve microbiological safety and extend the shelf life of products. This approach is gaining increasing interest among consumers who prefer food with a long shelf life and that is rich in vitamins and nutrients without the use of chemical preservatives [14,15].

The aim of the current study was to assess the quality and safety of hybrid meat products made from turkey meat and red beans with the addition of SAFEPRO^®^ B-LC-20 protective cultures.

## 2. Results

### 2.1. Chemical Composition (%)

**Figure 1:**
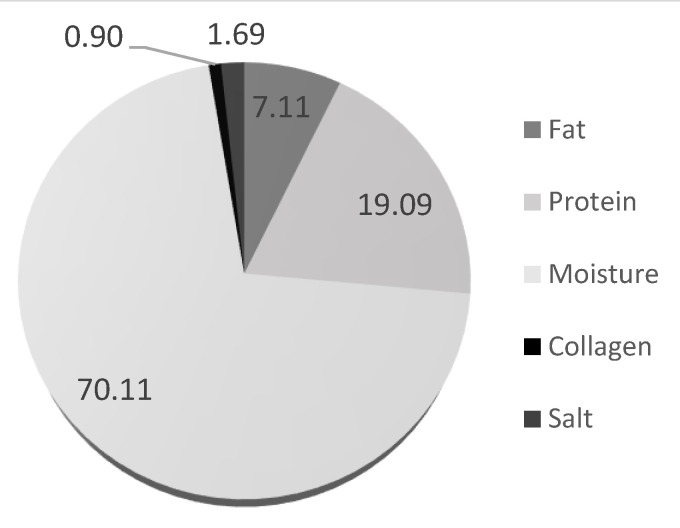
Chemical composition (%) of sample M100_B0—products produced with meat and red beans in the ratio of 100:0.

The chemical composition (%) of reference sample M100_B0 (products produced with meat and red beans in the ratio of 100:0) is shown in Figure 1. The average water, protein, fat, collagen and salt content percentages in the sample were 70.11%, 19.09%, 7.11%, 0.90%, and 1.69%, respectively.

### 2.2. Results of Microbiological Analyses

A statistical analysis of the microbiological test results showed a significant effect of the share of plant material and storage time on the count of *Enterobacteriaceae*, lactic acid bacteria and *E. coli* in hybrid meat products (Table 1). Throughout the storage period, samples with 40, 50 and 60% of red beans were characterized by a significantly higher count of lactic acid bacteria compared to the sample with 100% turkey meat. Up to day 8 of storage, no significant changes in lactic acid bacteria were observed compared to day 0, although significantly higher values (*p* ≤ 0.05) were observed on day 15 of storage. The count of *Enterobacteriaceae* did not differ significantly between samples on days 0, 8 and 15 of storage, although a significant increase in the count of these microorganisms was observed with the passage of storage time.

The count of *E. coli* on day 0 did not differ significantly between samples and ranged from 1.90 log CFU g^−1^ to 2.10 log CFU g^−1^. On days 8 and 15, a significant reduction in *E. coli* count was observed in all samples except for sample M40_B60. An analysis for the presence of *Listeria monocytogenes* and *Salmonella* spp. did not reveal the presence of these pathogens in the samples at the beginning or the end of the experiment. The absence of these pathogens is important in order to meet the microbiological criteria in accordance with Regulation (EC) No. 2073/2005.

### 2.3. pH Level and Water Activity (a_w_)

The measurements of physicochemical properties showed a significant effect (*p* ≤ 0.05) of the ratio of meat and plant material and storage time on the pH of hybrid meat products (Table 2). On days 0 and 8, the sample with the highest share of red beans (M40_B60) showed a significantly lower pH compared to the other samples. In the last period of storage (day 15), hybrid products with red beans were characterized by a significantly lower pH (by approximately 04–0.5 units) compared to the sample produced from 100% turkey meat (M100_B0). Water activity values did not differ significantly between the samples. No effect of storage time on this parameter was observed. The samples of the experimental products were characterized by an a_w_ ranging from 0.973 to 0.977.

### 2.4. Antioxidant Activity

The results of measuring the antioxidant activity of hybrid meat products showed statistically significant differences (*p* ≤ 0.05) between the formulations (Table 3). The addition of red beans significantly increased the antioxidant activity of the products compared to the M100_B0 sample. With the increase in the percentage of beans, an increase in antioxidant activity was observed against both the ABTS radical and DPPH. For the antioxidant capacity of the radical DPPH, no significant differences were found between the samples at different storage times. In terms of the measurement of antioxidant activity against the ABTS radical, the lowest values were observed on day 8 (0.051–0.087 mg Trolox eqv. g^−1^) and the highest on day 15 of storage (0.101–0.127 mg Trolox eqv. g^−1^).

The addition of beans resulted in significantly higher values of the substances reactive to 2-thiobarbituric acid (TBARS), indicating the increase in lipid oxidation compared to the product made from 100% meat (M100_B0). As the amount of plant material increased, the level of TBARS also rose. Additionally, its value increased significantly with the storage time for the red bean samples. TBARS was in the lowest range for sample M100_B0 (0.542–0.659 mg MDA kg^−1^) throughout the storage period and in the highest range for sample M40_B60 (1.182–1.640 mg MDA kg^−1^).

### 2.5. Lipid Quality Indicators

The main fractions of the fatty acid profile and lipid quality indicators of hybrid meat products are presented in Table 4. The addition of beans in amounts of 50 and 60% resulted in significantly higher values of ΣSFA as well as significantly lower values of the sum of PUFA. There were no significant differences between the samples in terms of MUFA. The proportion of n-3 acids in the fatty acid profiles of hybrid meat products was significantly lower (*p* ≤ 0.05) in formulations M50_B50 and M40_B60 than in M100_B0 and M60_B40. Similar relationships were observed for n-6 fatty acids. With the increase in the share of red beans, the content of n-6 decreased. Considering the value of lipid indicators in the hybrid meat products, there was a significant effect of formulation on n-6/n-3, UFA/SFA, PUFA/SFA, the atherogenicity index (AI) and the thrombogenicity index (TI). The samples with red beans were characterized by significantly lower values of n-6/n-3, UFA/SFA and PUFA/SFA compared to sample M100_B0. The formulations with 50 and 60% bean contents were characterized by significantly higher AI and TI lipid quality indicators.

### 2.6. Sensory Parameters

The findings of the sensory evaluation demonstrated significant differences between the analyzed products (Table 5). This includes all evaluated parameters except for color on the cross-section, which was rated similarly by the panelists for all hybrid products. In terms of juiciness and hardness, the highest scores were obtained for products with 100% and 60% turkey meat (M100_B0 and M60_B40), while significantly lower scores were obtained for products with 50% and 40% meat (M50_B50 and M40_B60). Similar relationships were obtained for the intensity of meat aroma and taste, while the opposite was true for the intensity of bean aroma and taste. The sample with 40% red beans (M60_B40) was characterized by low intensity of bean smell and taste (1.47 c.u. and 3.00 c.u., respectively). The samples with 50% and 60% beans obtained significantly higher scores in terms of bean aroma and taste intensity (5.50–7.10 c.u. and 6.79–7.75 c.u., respectively). The scores of individual sensory parameters translated into the overall quality of the products. Samples M100_B0 and M60_B40 obtained the highest scores in terms of overall quality. Interestingly, the score for the sample with 40% beans did not differ significantly from that of the sample with 100% meat. The samples with a higher red bean content obtained very low scores for overall quality (2.66–3.10 c.u.).

## 3. Discussion

In recent years, there has been a shift towards plant-based diets. A particularly large food consumer group are flexitarians, who do not eliminate meat consumption but only limit it in favor of plant-based foods [3]. In this context, the food industry is actively developing hybrid meat products to cater to this demand [6]. As stated by Grasso [16], designing hybrid foods by blending animal-based and plant-based ingredients aims to make food products that are similar to those made entirely from meat. In the current study, taking into account the determined chemical composition of the reference sample (products produced with 100% meat) and the chemical composition of the beans (declared by the manufacturer), it can be assumed that with the increase in the share of beans in the formulation, the fat and protein content will decrease in the hybrid meat product. The results obtained by Sun et al. [17], who investigated the impact of hempseed meal at 10%, 20%, 30%, and 40% inclusion on the quality characteristics of chicken sausage, indicated that hempseed meal decreased the protein and lipid content. Research conducted by Chandler and McSweeney [18] on the properties of hybrid meat burgers made with legume flours (yellow pea, chickpea and lentil) and chicken showed that the use of yellow pea resulted in a reduced protein content.

A relevant topic to be considered in hybrid product design is limiting the number of ingredients and additives employed to replicate meat-like characteristics. A large number of additives, including those protecting against spoilage, may be negatively perceived by consumers and become a barrier, especially for those interested in clean labels and nutrition-related issues [19]. In this study, additives were eliminated in line with the “clean label” trend [20]. Only sodium nitrate was used, but in an amount three times lower than the limit specified in food law [21]. The composition of the curing salt enabled the introduction of 50 mg/kg of sodium nitrite into the product. For this reason, bioprotective cultures were used to improve microbiological safety and extend the shelf life of the products [15]. Commercial cultures containing *Pediococcus acidilactici* strains were used. The effectiveness of *Pediococcus acidilactici* strains and their bacteriocins in meat products has been demonstrated [22,23]. The studies of Slima et al. [22] on the evaluation of the effects of a combination of *Lactobacillus plantarum* TN8 and *Pediococcus acidilactici* MA 18/5M found a decrease in the count of *Enterobacteriaceae* in beef sausages. The current study observed that the count of *Enterobacteriaceae* in hybrid meat products did not differ significantly depending on the formulation. At the same time, due to the use of protective cultures, a high content of lactic acid bacteria was observed. Antagonistic effects of protective cultures were observed in relation to *E. coli* bacteria. The count of *E. coli* decreased with the storage time of hybrid meat products elapsed.

Red beans appear to be a valuable ingredient in hybrid meat products not only as a significant source of vegetable protein, fiber, and certain micronutrients but also due to their wide variety of bioactive compounds [24]. Colored beans are a significant source of phenolic compounds; more specifically, phenolic acids, flavonoids, and anthocyanidins, which are the main phenolic compounds identified and characterized in beans [25]. Research conducted by Martínez-Alonso et al. [24] showed that epicatechin and delphinidin were the most detected polyphenols found in red bean extracts. Moreover, Madhujith et al. [26] demonstrated that beans possess strong antioxidant activity as measured by different model systems. The current study confirmed that the addition of red beans significantly increased the antioxidant activity of hybrid meat products. It was indicated that with the increase in the percentage of beans, an increase in antioxidant activity was observed against both the ABTS radical and DPPH. The antioxidant properties did not influence the TBARS values related to the number of substances that react with thiobarbituric acid, which includes secondary products of fat oxidation. It was observed that with the increase in the share of red beans in the formulation of hybrid products, the TBARS value increased. Additionally, its value increased significantly with the storage time for the red bean samples. Generally, thiobarbituric-acid-reactive substances (TBARSs) reflect the byproducts resulting from lipid peroxidation in food matrix. The major end products of lipid peroxidation are reactive aldehydes, such as malondialdehyde (MDA) and 4-hydroxynonenal. However, sometimes, TBARS test results are difficult to interpret because MDA is not the only one that can react with thiobarbituric acid under the test conditions. The overestimation of MDA can also be due to the fact that other types of compounds (carbohydrates, amino acids and nucleic acids) can react with TBA and absorb at 532 nm, as indicated by some studies [27]. The TBARS values for all samples in the current study were at a low level and did not exceed the value of 2.0 mg kg^–1^, which is the most frequently indicated threshold value for meat products’ rancidity.

In the context of oxidation processes, both the antioxidant properties and the profile of fatty acids are very important. It is well known that unsaturated and especially polyunsaturated fatty acids are first oxidized to form odorless and tasteless hydroperoxides, which are further degraded to form secondary oxidation products [28]. An analysis of the main fractions of the fatty acid profile indicated that the hybrid product formulation with the addition of beans in amounts of 50% and 60% resulted in significantly higher values of the sum of SFA, as well as significantly lower values of the sum of PUFA. Similarly, a decrease in the content of n-3 fatty acids was found with the increase in the addition of red beans. Only the formulation with meat and beans in a ratio of 40:60 did not cause changes in the content of individual fatty acid fractions compared to the formulation with 100% turkey meat.

Considering the value of lipid quality indicators of hybrid meat products, the formulation had a significant impact on the assessed indicators. When the share of red beans in the formulation of hybrid products was 50% and 60%, an increase in PUFA/SFA was observed. Formulations of hybrid meat products with 100% and 60% turkey meat were characterized by an average indicator PUFA/SFA in the range of 0.42–0.51. This is positive for the consumer, as this is close to the recommended value for preventing ischemic heart disease (0.45) [29]. Hybrid products with a higher share of red beans were characterized by an unfavorable index PUFA/SFA (below 0.36). Red bean inclusion resulted in a significant reduction in the n-6/n-3 ratio of hybrid meat products, which is beneficial from a nutritional point of view. Baune et al. [30] replaced 30% of pork meat with three types of protein (from pea, sunflower or pumpkin) in two different forms (either wet or dry extruded textured protein) in a hybrid meat product. Their results, similar to those of the current study, showed that replacing meat with plant protein improved the ω-6/ω-3 ratio (approximately 8:1 in comparison to controls 12:1) in hybrid meat products. As noted by Ulbricht and Southgate [31], n-6 PUFAs are correlated with the reduction in serum lipids, while n-3 PUFAs are associated with the inhibition of platelet aggregation and the reduction in cholesterol and phospholipid levels. The AI index also indicates that meat products made with meat and red beans in the ratios 100:0 and 60:40 are the most beneficial in terms of the nutritional quality of lipids. AI is lower in these formulations compared to that in 50:50 and 40:60 formulations, which indicates that the former formulations offer the more favorable fatty acid profile. Ouraji et al. [32] reported that an AI index above 1.0 is harmful to humans. In the current study, all samples obtained an AI index below 1.0. However, another index, i.e., TI, whose value <1.0 is also recommended, had values higher than 1.0.

According to Baune et al. [33], hybrid meat products with a high substantial meat substitution level often fail in the market. In this context, a sensory evaluation of the products is essential for developing new hybrid meat products and understanding consumer expectations [34]. When developing a formulation for a new hybrid meat product, it is crucial to focus on texture and flavor, the main attributes that influence consumer liking. As stated by Verbeke [35], since consumers are not willing to compromise their taste for health, hybrid meat products must match 100% meat products concerning sensory characteristics. In the current study, the findings for the sensory evaluation demonstrate that hybrid products with meat and red beans in the ratios of 60:40 were characterized by very similar scores in terms of juiciness, hardness, intensity of meat flavor and odor, as well as overall quality. The hybrid meat products with a higher red bean content obtained very low scores for overall quality (2.66–3.10 c.u.), which indicates that their quality greatly changed compared to that of a 100% meat product. Similarly, research conducted by Bakhsh et al. [36] on the effect of different levels of textured vegetable protein substitution on the quality of beef patties showed reduced hardness, cohesiveness, and thickness, with increased gumminess and chewiness when soy-based textured vegetable protein was applied at the level of 10–40%. Additionally, the Electronic Tongue System showed a higher tendency for sourness, astringency, umami, and saltiness values of hybrid beef patties with soy proteins.

## 4. Materials and Methods

### 4.1. Preparation of the Experimental Material

**Figure 2:**
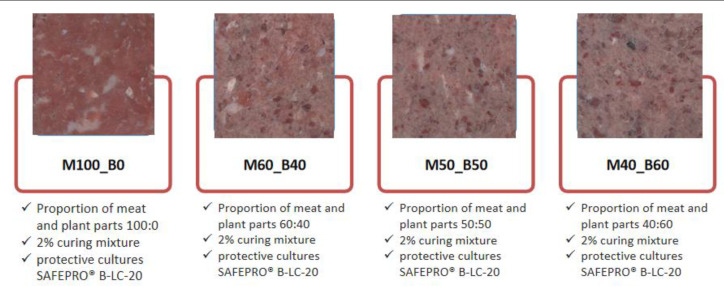
The experimental design, compositions and cross-section appearance of hybrid meat products.

Hybrid meat products produced with turkey thigh muscles and red beans in ratios of 100:0 (M100_B0), 60:40 (M60_B40), 50:50 (M50_B50) and 40:60 (M40_B60) were used as the experimental material. Turkey thigh muscles were obtained from Stasin Poultry Pant (Stasin, Poland), and canned red beans (Ronik, Mikołów, Poland) were purchased in a local store. According to the manufacturer’s declaration, the nutritional value of beans in 100 g of the product was as follows: fat 0.5 g; carbohydrates 15 g; fiber 5.7 g; protein 7.7 g. An additional ingredient was a curing mixture (salt + sodium nitrite; 2%). The composition of the curing salt enabled the introduction of 50 mg/kg of sodium nitrite into the product. Protective cultures (SAFEPRO^®^ B-LC-20, Chr. Hansen) were used to ensure the safety and shelf life of the products. As stated by the manufacturer, the cultures contained *Pediococcus acidilactici*. The meat and red beans were ground using a grinder (KU2-3EK, Mesko-AGD Skarżysko-Kamienna, Poland) using a plate with 0.005 m holes. All ingredients were mixed using a universal machine (KU2-3EK, Mesko-AGD, Skarżysko-Kamienna, Poland) with an attached R4 agitator (100 rpm for 3 min). Approximately 100 g ± 10 g of stuffing was then formed into meatballs. In this way, raw samples of hybrid meat products in the form of meatballs were obtained. The experimental design and compositions of meat product samples are shown in Figure 2. Samples were taken for analysis immediately after preparation (day 0). The samples were then vacuum-packed and stored for 15 days. During storage, analyses were carried out after 8 and 15 days. Two batches were produced for each treatment.

### 4.2. Chemical Composition Analysis

Composition analysis was performed using a Food Scan Lab 78,810 (Foss Tecator Co., Ltd., Hillerod, Denmark). Approximately 200 g of a sample was distributed in the instrument’s round sample dish and loaded into the instrument’s sample chamber. The collagen, moisture, protein, salt and fat contents were determined.

### 4.3. Microbiological Analyses

The counts of lactic acid bacteria, *Enterobacteriaceae*, and *Escherichia coli* were analyzed using the TEMPO^®^ LAB automated microbial counting system (Biomerieux, TEMPO^®^ System, Marcyl’Etoile, France). The original TEMPO^®^ tests were used to determine the count of lactic acid bacteria (TEMPO LAB), *Enterobacteriaceae* (TEMPO EB) and *Escherichia coli* (TEMPO EC) in hybrid meat products. The incubation conditions used for the TEMPO LAB, TEMPO EB and TEMPO EC tests were as follows: incubation time 40–48 h (LAB), 22–27 h (EB, EC); and temperature of incubation of 37 °C (LAB) and 35 ^°^C (EB, EC). The results are expressed as a log CFU g^−1^. Additionally, *L. monocytogenes* and *Salmonella* spp. detection was carried out at Agrolab Polska Sp. z o. o. (Dęblin, Poland) according to ISO 11290-1:2017 [37] and ISO 6579-1:2017-04 [38], respectively.

### 4.4. Water Activity (a_w_) and pH Level

The water activity (a_w_) was measured using a water activity analyzer (Novasina AG, Lachen, Switzerland). Measurements were carried out at a temperature of 20 °C. The analyzer was calibrated with Novasina SAL-T humidity standards (33%, 75%, 84%, and 90% relative humidity). For pH measurement, a digital pH meter CPC-501 (Elmetron, Zabrze, Poland) equipped with a temperature sensor and pH electrode (ERH-111, Hydromet, Gliwice, Poland) was used. The pH measurement was performed on a homogenate of the hybrid product sample and distilled water (1:5 ratio) prepared just before the measurement.

### 4.5. Determination of Antioxidant Capacity (DPPH and ABTS Methods) and TBARS Values

The antioxidant capacity of the radical 2,2-diphenyl-1-picrylhydrazyl (DPPH) and 2,20-azino-bis(3-ethylbenzothiazoline-6-sulphonic acid) diammonium salt was carried out according to a previous method [39]. The sample’s ability to scavenge ABTS*+ and DPPH free radicals was assessed by comparing the results to a Trolox standard curve. The results were expressed in milligrams per gram of the product.

The lipid oxidation process was analyzed using the method for measuring substances reactive to 2-thiobarbituric acid (TBARS). The quantification of compounds reacting with thiobarbituric acid was conducted following the procedure described by Pikul et al. [40], with perchloric acid used as the solvent. Absorbance was measured at 532 nm using a UV spectrophotometer (Nicolet Evolution 300, Thermo Electron Corp., Waltham, MA, USA). The results were presented as mg of malondialdehyde (MDA) per kilogram of sample.

### 4.6. Fatty Acid Profile Measurements

Fatty acid (FA) extraction and analysis were carried out using a gas chromatograph (GC-Agilent7890B, Agilent Technologies, Santa Clara, CA, USA) following the conditions reported by Barros et al. [41]. Individual fatty acid groups, as well as the atherogenic index (AI) and thrombogenic index (TI), were calculated according to the work of Goluch et al. [29] (Table 6).

### 4.7. Sensory Evaluation

The sensory quality of hybrid meat products was assessed using descriptive analysis with a 10-point linear scale ranging from 1 to 10 units (c.u.), according to ISO 13299:1998 (QDA) [42]. The raw hybrid products were heat-treated in a water bath at 85 °C ± 3 °C until a core temperature of 72 °C was reached and then cooled to room temperature (21 °C ± 2 °C). The cooled products were placed in transparent, odorless plastic containers with lids. Each sample was individually coded with three digits and presented randomly to minimize transfer effects. The evaluation was conducted by ten trained panelists. Consistent temperature, lighting, and elimination of distracting factors such as noise and unpleasant odors were maintained during the analysis. The parameters evaluated and their descriptors were as follows: color in cross-section (from gray to very red), juiciness (from dry to very juicy), hardness (from low to very high), meat aroma intensity (from imperceptible to very intense), bean aroma intensity (from imperceptible to very intense), meat taste intensity (from imperceptible to very intense), bean taste intensity (from imperceptible to very intense), and overall quality (from poor to very good).

### 4.8. Statistical Analysis

The data collected during the study were processed using Statistica software version 9.1 (StatSoft, Poland) and presented as mean ± standard deviation. All measurements were performed with at least three replicates. The normality of the variable distributions in the study groups was assessed using the Shapiro–Wilk test. Data analysis was conducted using a two-way analysis of variance (ANOVA). Differences between mean values were determined using Tukey’s test at *p* < 0.05.

## 5. Conclusions

Based on the findings, the amount of red beans in the formulation of hybrid meat products has a significant impact on the physicochemical characteristics, antioxidant properties, nutritional value and sensory characteristics. With the increase in the share of red beans in the product’s formulation, a less favorable fatty acid profile and lipid quality indicators were observed, as well as altered sensory parameters compared to the 100% meat product. However, hybrid products with a higher share of plant material showed a higher antioxidant activity. The sample made from meat and red beans in a ratio of 60:40 was the most similar to the sample made from 100% meat in terms of the assessed properties. In summary, the formulation with turkey meat and beans in a ratio of 60:40 is recommended as optimal as this combination produces a safe hybrid meat product with properties similar to that of a full-meat product. The present study allowed for the development of hybrid meat product formulations (with turkey meat and beans in a ratio of 60:40) that could potentially meet consumer expectations. Further studies should focus on this formulation and include in-depth analyses of bioactive properties and storage, and consumer studies.

## Figures and Tables

**Table 1:** The results of microbiological analyses of hybrid meat products.

		M100_B0	M60_B40	M50_B50	M40_B60
*Enterobacteriaceae* [log CFU g^−1^]	Day 0	3.20 ± 0.11 ^aA^	3.13 ± 0.16 ^aA^	3.13 ± 0.18 ^aA^	3.09 ± 0.21 ^aA^
	Day 8	5.40 ± 0.33 ^aB^	5.36 ± 0.57 ^aB^	5.69 ± 0.30 ^aB^	4.99 ± 0.44 ^aB^
	Day 15	7.59 ± 0.63 ^aC^	6.95 ± 0.28 ^aC^	6.88 ± 0.31 ^aC^	6.80 ± 0.24 ^aC^
*Lactic acid bacteria* [log CFU g^−1^]	Day 0	7.72 ± 0.25 ^aA^	8.18 ± 0.21 ^bA^	8.05 ± 0.20 ^bA^	8.42 ± 0.26 ^bA^
	Day 8	7.31 ± 0.54 ^aA^	8.09 ± 0.26 ^bA^	8.11 ± 0.29 ^bA^	8.17 ± 0.17 ^bA^
	Day 15	8.06 ± 0.50 ^aA^	9.28 ± 0.05 ^bB^	9.41 ± 0.03 ^bB^	9.57 ± 0.31 ^bB^
*E. coli* [log CFU g^−1^]	Day 0	2.04 ± 0.12 ^aB^	2.04 ± 0.15 ^aA^	2.10 ± 0.10 ^aB^	1.90 ± 0.35 ^aA^
	Day 8	1.71 ± 0.27 ^aA^	1.97 ± 0.28 ^aA^	1.67 ± 0.28 ^aA^	1.77 ± 0.24 ^aA^
	Day 15	1.40 ± 0.25 ^aA^	1.90 ± 0.19 ^bA^	1.78 ± 0.16 ^bA^	1.75 ± 0.15 ^bA^
*L. monocytogenes* [CFU g^−1^]	Day 0	<10	<10	<10	<10
	Day 15	<10	<10	<10	<10
*Salmonella* spp. [CFU g^−1^]	Day 0	<10	<10	<10	<10
	Day 15	<10	<10	<10	<10

M100_B0—products produced with meat and red beans in the ratio of 100:0; M60_B40—products produced with meat and red beans in the ratio of 60:40; M50_B50—products produced with meat and red beans in the ratio of 50:50; M40_B60—products produced with meat and red beans in the ratio of 40:60. Different lowercase letters (a–b) between means in rows indicate significant differences at *p* ≤ 0.05. Different uppercase letters (A–C) between means in columns indicate significant differences.

**Table 2:** The results of water activity (a_w_) and pH level measurements of hybrid meat products.

		M100_B0	M60_B40	M50_B50	M40_B60
pH	Day 0	5.95 ± 0.11 ^bA^	5.93 ± 0.01 ^bB^	5.91 ± 0.02 ^bB^	5.79 ± 0.03 ^aB^
	Day 8	5.94 ± 0.02 ^cA^	5.88 ± 0.01 ^bB^	5.88 ± 0.04 ^bB^	5.81 ± 0.02 ^aB^
	Day 15	5.95 ± 0.06 ^bA^	5.39 ± 0.04 ^aA^	5.45 ± 0.09 ^aA^	5.55 ± 0.11 ^aA^
a_w_	Day 0	0.976 ± 0.002 ^aA^	0.975 ± 0.002 ^aA^	0.973 ± 0.003 ^aA^	0.977 ± 0.001 ^aA^
	Day 8	0.974 ± 0.006 ^aA^	0.974 ± 0.006 ^aA^	0.977 ± 0.005 ^aA^	0.976 ± 0.006 ^aA^
	Day 15	0.975 ± 0.003 ^aA^	0.974 ± 0.004 ^aA^	0.976 ± 0.005 ^aA^	0.976 ± 0.004 ^aA^

M100_B0—products produced with meat and red beans in the ratio of 100:0; M60_B40—products produced with meat and red beans in the ratio of 60:40; M50_B50—products produced with meat and red beans in the ratio of 50:50; M40_B60—products produced with meat and red beans in the ratio of 40:60. Different lowercase letters (a–c) between means in rows indicate significant differences at *p* ≤ 0.05. Different uppercase letters (A–B) between means in columns indicate significant differences.

**Table 3:** Antioxidant activity and TBARS values of hybrid meat products.

		M100_B0	M60_B40	M50_B50	M40_B60
ABTS [mg Trolox eqv. g^−1^]	Day 0	0.078 ± 0.007 ^aB^	0.091 ± 0.005 ^bB^	0.096 ± 0.005 ^bB^	0.099 ± 0.004 ^bB^
	Day 8	0.051 ± 0.005 ^aA^	0.072 ± 0.005 ^bA^	0.079 ± 0.003 ^bA^	0.087 ± 0.003 ^cA^
	Day 15	0.101 ± 0.005 ^aC^	0.107 ± 0.001 ^bC^	0.114 ± 0.004 ^cC^	0.127 ± 0.002 ^dC^
DPPH [mg Trolox eqv. g^−1^]	Day 0	0.133 ± 0.005 ^aA^	0.150 ± 0.002 ^bA^	0.153 ± 0.003 ^bA^	0.155 ± 0.002 ^bA^
	Day 8	0.138 ± 0.003 ^aA^	0.147 ± 0.004 ^bA^	0.152 ± 0.002 ^bA^	0.155 ± 0.004 ^bA^
	Day 15	0.137 ± 0.004 ^aA^	0.146 ± 0.002 ^bA^	0.149 ± 0.003 ^bA^	0.152 ± 0.005 ^bA^
TBARS [mg MDA kg^−1^]	Day 0	0.547 ± 0.025 ^aA^	0.968 ± 0.092 ^bA^	1.178 ± 0.102 ^cA^	1.182 ± 0.091 ^cA^
	Day 8	0.659 ± 0.026 ^aB^	1.262 ± 0.231 ^bB^	1.352 ± 0.193 ^bB^	1.351 ± 0.127 ^bB^
	Day 15	0.542 ± 0.029 ^aA^	1.123 ± 0.113 ^bB^	1.436 ± 0.208 ^cB^	1.640 ± 0.134 ^cC^

M100_B0—products produced with meat and red beans in the ratio of 100:0; M60_B40—products produced with meat and red beans in the ratio of 60:40; M50_B50—products produced with meat and red beans in the ratio of 50:50; M40_B60—products produced with meat and red beans in the ratio of 40:60. Different lowercase letters (a–c) between means in rows indicate significant differences at *p* ≤ 0.05. Different uppercase letters (A–C) between means in columns indicate significant differences.

**Table 4:** Main fractions of the fatty acid profile [%] and lipid quality indicators of hybrid meat products.

	M100_B0	M60_B40	M50_B50	M40_B60
Σ SFA	36.63 ± 0.92 ^a^	37.72 ± 0.54 ^a^	40.39 ± 0.04 ^b^	41.33 ± 4.92 ^b^
Σ MUFA	37.36 ± 0.24 ^a^	38.02 ± 0.36 ^a^	38.65 ± 0.17 ^a^	37.26 ± 2.33 ^a^
Σ PUFA	18.59 ±1.87 ^b^	15.67 ± 1.69 ^b^	10.71 ± 0.31 ^a^	12.49 ± 2.49 ^a^
Σ n-3	1.21 ± 0.11 ^b^	1.15 ± 0.12 ^b^	0.84 ± 0.11 ^a^	0.81 ±0.12 ^a^
Σ n-6	17.38 ± 1.76 ^c^	14.52 ± 1.57 ^b^	9.87 ± 0.30 ^a^	11.68 ± 2.11 ^a^
n-6/n-3	14.39 ± 0.22 ^b^	12.62 ± 0.07 ^a^	11.75 ± 0.24 ^a^	12.51 ± 2.64 ^a^
UFA/SFA	1.53 ± 0.09 ^c^	1.42 ± 0.06 ^c^	1.22 ± 0.01 ^a^	1.36 ± 0.03 ^b^
PUFA/SFA	0.51 ± 0.06 ^c^	0.42 ± 0.05 ^c^	0.27 ± 0.01 ^a^	0.36 ±0.02 ^b^
AI	0.69 ± 0.04 ^a^	0.74 ± 0.03 ^ab^	0.86 ± 0.01 ^c^	0.77 ± 0.02 ^b^
TI	1.12 ± 0.07 ^a^	1.20 ± 0.06 ^ab^	1.43 ± 0.01 ^c^	1.26 ± 0.01 ^b^

M100_B0—products produced with meat and red beans in the ratio of 100:0; M60_B40—products produced with meat and red beans in the ratio of 60:40; M50_B50—products produced with meat and red beans in the ratio of 50:50; M40_B60—products produced with meat and red beans in the ratio of 40:60. Different lowercase letters (a–c) between means in rows indicate significant differences at *p* ≤ 0.05.

**Table 5:** Sensory parameters of hybrid meat products.

	M100_B0	M60_B40	M50_B50	M40_B60
Color in cross-section	8.43 ± 1.36 ^a^	7.64 ± 1.90 ^a^	7.50 ± 1.66 ^a^	6.25 ± 2.25 ^a^
Juiciness	5.43 ± 1.04 ^b^	5.22 ± 1.37 ^b^	2.38 ± 1.44 ^a^	1.83 ± 1.31 ^a^
Hardness	6.00 ± 2.06 ^b^	5.58 ± 0.49 ^b^	3.07 ± 1.24 ^a^	1.58 ± 0.86 ^a^
Intensity of meat aroma	7.97 ± 1.19 ^c^	7.36 ± 1.17 ^c^	5.56 ± 1.04 ^b^	2.50 ± 1.54 ^a^
Intensity of bean aroma	0.14 ± 0.21 ^a^	1.47 ± 1.35 ^b^	5.50 ± 1.73 ^c^	7.10 ± 1.43 ^d^
Intensity of meat taste	8.46 ± 1.8 ^b^	6.98 ± 1.16 ^b^	3.93 ± 1.34 ^a^	3.48 ± 1.83 ^a^
Intensity of bean taste	0.11 ± 0.15 ^a^	3.00 ± 1.53 ^b^	6.79 ± 1.75 ^c^	7.75 ± 1.41 ^c^
Overall quality	8.11 ± 1.75 ^b^	7.14 ± 1.34 ^b^	3.10 ± 1.75 ^a^	2.66 ± 0.59 ^a^

M100_B0—products produced with meat and red beans in the ratio of 100:0; M60_B40—products produced with meat and red beans in the ratio of 60:40; M50_B50—products produced with meat and red beans in the ratio of 50:50; M40_B60—products produced with meat and red beans in the ratio of 40:60. Different lowercase letters (a–d) between means rows indicate significant differences at *p* ≤ 0.05.

**Table 6:** Calculation of fatty acid groups and lipid quality indicators [29].

Fatty Acid Group/Lipid Quality Indicators	Calculation Formula
ΣSFA (Saturated fatty acids)	Sum from C4:0 to C24:0
ΣMUFA (Monounsaturated fatty acids)	Sum from C14:1 to C24:1
ΣPUFA (Polyunsaturated fatty acids)	Σ n-3 PUFA + Σ n-6 PUFA
ΣPUFA n-3 (Polyunsaturated fatty acids n-3)	C18:3 n-3 + C18:4 n-3 + C20:3 n-3 + C20:5 n-3 + C22:5 n-3 + C22:6 n-3
ΣPUFA n-6 (Polyunsaturated fatty acids n-6)	C18:2 n-6 + C18:3 n-6 + C20:3 n-6 + C20:4 n-6 + C22:2 n-6 + C22:4 n-6
ΣPUFA n-3/ΣPUFA n-6	C18:3 n-3 + C18:4 n-3 + C20:3 n-3 + C20:5 n-3 + C22:5 n-3 + C22:6 n-3/C18:2 n-6 + C18:3 n-6 + C20:3 n-6 + C20:4 n-6 + C22:2 n-6 + C22:4 n-6
ΣUFA/ΣSFA Unsaturated/Saturated fatty acids	ΣMUFA + ΣPUFA//ΣSFA
ΣPUFA/ΣSFA Polyunsaturated/Saturated fatty acids	Σ n-3 PUFA + Σ n-6 PUFA/ΣSFA
AI Atherogenicity index	[C12:0 + (4 × C14:0) + C16:0 + C18:0]/[Σ MUFA + Σ PUFA n-6 + Σ PUFA n-3
TI Thrombogenicity index	(C14:0 + C16:0 + C18:0)/[(0.5 × Σ MUFA) + (0.5 × Σ PUFA n-6) + (3 × Σ PUFA n-3) + (Σ PUFA n-3/ΣPUFA n-6)]

## Data Availability

The original contributions presented in this study are included in this article. Further inquiries can be directed to the corresponding author.
